# Human to Mosquito Transmission of Dengue Viruses

**DOI:** 10.3389/fimmu.2014.00290

**Published:** 2014-06-17

**Authors:** Lauren B. Carrington, Cameron P. Simmons

**Affiliations:** ^1^Dengue Group, Oxford University Clinical Research Unit, Hospital for Tropical Diseases, Ho Chi Minh City, Vietnam; ^2^Department of Microbiology and Immunology, The University of Melbourne, Melbourne, VIC, Australia; ^3^Nuffield Department of Clinical Medicine, Centre for Tropical Medicine, University of Oxford, Oxford, UK

**Keywords:** dengue virus, transmission, *Aedes aegypti*, *Aedes albopictus*, viral titer, temperature, symptomatic vs. asymptomatic infections

## Abstract

The successful transmission of dengue virus from a human host to a mosquito vector requires a complex set of factors to align. It is becoming increasingly important to improve our understanding of the parameters that shape the human to mosquito component of the transmission cycle so that vaccines and therapeutic antivirals can be fully evaluated and epidemiological models refined. Here we describe these factors, and discuss the biological and environmental impacts and demographic changes that are influencing these dynamics. Specifically, we examine features of the human infection required for the mosquito to acquire the virus via natural blood feeding, as well as the biological and environmental factors that influence a mosquito’s susceptibility to infection, up to the point that they are capable of transmitting the virus to a new host.

## Introduction

### The global dengue burden

The medical (1) and economic (2–7) burden of dengue is large; a recent probabilistic estimate suggested 100 million symptomatic cases occurred in 2010 (8). Human travel patterns are changing, and there is far more international traffic between dengue endemic countries and those that are usually dengue-free, albeit permissive for epidemics because of the presence of a suitable vector (9–11). This is evidenced by recent autochthonous dengue virus (DENV) transmission in Europe (12, 13) (local transmission subsequent to importation). The current scale of the public health problem of dengue highlights the need to better understand the underlying biological and environmental factors that result in human to mosquito transmission of DENV. A better comprehension of how these factors vary, and under what conditions, will help us to develop more effective interventions of DENV transmission.

### Human to mosquito transmission of dengue

Transmission of DENV from the human host to mosquitoes requires multiple biological factors to align in time and space. Under natural conditions, a susceptible mosquito can only acquire a DENV infection after it has taken a blood meal from a viremic person. When viremic blood arrives into the mosquito midgut, extracellular virus binds to undefined receptors on the cellular surface of the midgut epithelium. If the virus can successfully infect and replicate within midgut epithelium cells then new progeny virus are shed into the hemocoel (the cavity in which the hemolymph circulates, part of the open circulatory system of invertebrates), where it can subsequently disseminate and infect secondary tissues, including the salivary glands. Once sufficient virus replication has occurred in the salivary glands and upon the next probing/feeding event, the virus may be transmitted to a new host via the saliva of the infected mosquito.

### Vectors of DENV

The primary vector of DENV is *Aedes aegypti*, an endophilic mosquito, preferring to live in and around homes in tropical and subtropical regions. This mosquito feeds preferentially on human blood under field conditions (14), and inhabits tropical and subtropical climates, with the geographic range spanning all continents except Antarctica. A secondary dengue vector, *Aedes albopictus*, is more exophilic under natural field conditions, commonly living outdoors, but still feeds almost exclusively on humans in Thailand (14), and preferentially on humans in the Indian Ocean (15). The strong preferences for human blood exhibited by these mosquitoes increase the potential for disease transmission among humans.

The expanding geographical range of DENV vectors (16, 17) underscores our need to examine DENV transmission dynamics in more detail. In the United States there has been a resurgence of *Aedes aegypti* across the South Eastern seaboard, and its presence has been noted as far north in California as South San Francisco Bay (W. K. Reisen and M. V. Armijos, UC Davis, personal communication, August 2013). *Aedes aegypti* is also expected to spread beyond its current range within Australia, which is presently throughout the state of Queensland, extending into the North Eastern part of Northern Territory (18). Autochthonous cases of dengue occurred in Portugal (Madeira Islands) in 2012 (13), with transmission attributed to the invasion of *Aedes aegypti* in the mid 2000s.

*Aedes albopictus*, an aggressive, nuisance day-time biter (19), is now established in numerous areas of Southern Europe (20–23), with its geographic range having continuously expanded since its first observation in Albania in 1979 (24). *Aedes albopictus* has also become established in parts of South America and Africa that were previously free of the invasive pest (16) (and references therein). Its emergence in Australia is also a significant threat (25). This (potential and actual) range expansion of *Aedes albopictus*, particularly because it inhabits a more temperate environment than the tropical *Aedes aegypti*, may lead to an increased risk of DENV transmission as it brings a greater number of dengue-susceptible people into contact with vectors. Photoperiod-induced diapause and non-desiccating, cold-tolerant eggs further allows *Aedes albopictus* to survive in cooler environments for periods of the year (26, 27).

Other Aedine species have been shown to be capable of transmitting DENV under experimental conditions (28–30), including *Aedes polynesiensis, Aedes scutellaris*, and *Aedes japonicas*. As discussed in Rosen et al. (28), *Aedes polynesiensis* has been implicated in the natural transmission of DENV, but the relative contribution of each of these mosquitoes to overall transmission has not been quantified, and is thought to be negligible (31).

## Human Factors Influencing Transmission

Factors that influence the transmission of DENV from humans to mosquitoes include the following.

### Viral titer in human plasma

**Figure 1:**
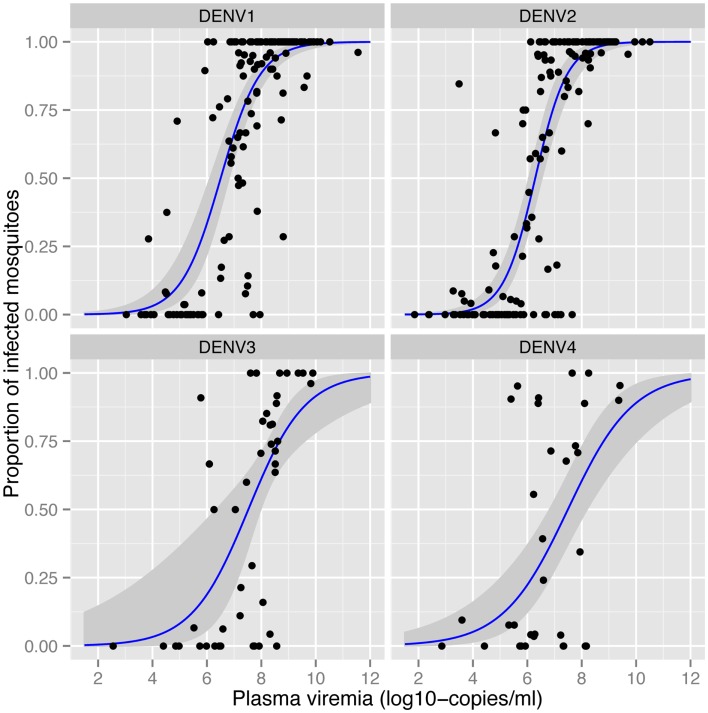
**Effect of plasma viremia on mosquito susceptibility to infection (32)**. With an increasing concentration of DENV in the patient’s blood, mosquitoes have a higher probability of being infected, as determined 12 days after mosquitoes imbibed the blood meal. Each data point represents the proportion of DENV-infected mosquitoes after a single blood-feeding episode. Estimated associations and the 95% confidence intervals are shown in the blue lines and gray shading, respectively. Image reproduced with permission from the authors.

The amount of virus circulating in the blood of an infected human will influence the likelihood of a mosquito becoming infected after a blood meal. Nguyen et al. (32) identified the viremia characteristics in dengue cases that led to DENV infection of blood-fed *Aedes aegypti*. The viremia in humans required to infect 50% of mosquitoes differed between serotypes (Figure 1). The 50% mosquito infectious dose was ~10-fold lower for DENV-1 and DENV-2 (6.29–6.51 log10 RNA copies/ml) than for DENV-3 and DENV-4 (7.49–7.52 log10 RNA copies/ml). A dose–response relationship was observed such that with an increasing number of DENV RNA copies, there was an increased likelihood of a mosquito becoming infected, up to the point of saturation. These findings define the viremia level that interventions such as vaccines and antivirals must target for prevention or amelioration to reduce DENV transmission.

### Duration of human infectiousness

**Figure 2:**
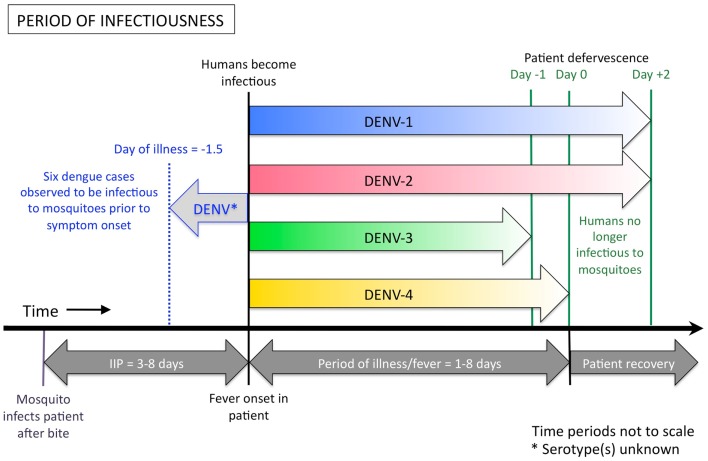
**Duration of human DENV infectiousness to *Aedes aegypti* mosquitoes**. A person can become infectious to mosquitoes up to 1.5 days before the onset of their fever and associated symptoms, and remain infectious until the end of their febrile period, and sometimes shortly after. Indicated above the thick black line are the colored arrows showing the period of human infectiousness for each serotype, according to Nguyen et al. (32). DENV-1 (shown in blue) and DENV-2 (red) may be infectious to mosquitoes for up to 2 days after the patient becomes afebrile, however DENV-3 (green) and DENV-4 (yellow) appear to be less infectious at these later stages of illness, due to lower plasma viremia in the patient. A single study (37), found that six dengue patients were infectious to mosquitoes from 0.25 days, up to a maximum of 1.5 days, before they had any sign of symptoms (indicated by the pale gray arrow). The serotype of virus used in Siler et al.’s study is unknown. Below the black line is the relative alignment of the course of dengue illness. IIP = intrinsic incubation period.

Accumulated data from empirical infection studies on human subjects conducted in the first half of the twentieth century showed that humans can be infectious to mosquitoes from 1.5 days prior to the onset of symptoms to around 5 days after the commencement of symptoms (Figure 2) (33–37). In each of these studies, however, the assignment of the day or hour of the mosquito exposures was not systematic [e.g., Cleland et al. (34) exposed mosquitoes to patients on the 18th, 22nd, 46th, 47th, 57th, 67th, and 90th hours after the onset of fever (with no apparent pattern or rationale behind the selection of these time points)], resulting in a broad range of exposure time points but with large gaps in between. In Nguyen et al.’s (32) more recent study, 208 patients who presented to the Hospital for Tropical Diseases in Ho Chi Minh City, Vietnam were enrolled in the study and randomly assigned to 2 days on which they would be exposed to naïve mosquitoes. Days of exposure ranged between day 1 and day 7 of illness. Results demonstrate that a small number of mosquitoes can still become infected with each of the four DENV serotypes up to the sixth day after illness onset. No mosquitoes became infected after feeding on patients on the seventh day after onset. Nguyen et al. (32) further demonstrated that patients with DENV-1 and DENV-2 infections can still be infectious to mosquitoes up to 2 days after defervescence, albeit this was rare. For patients infected with DENV-3 and DENV-4, viremia had declined below the required infectious dose for mosquitoes to become infected by this time. In addition, Nguyen et al. (32) demonstrated that patients with a high early viremia have a greater probability of having an extended duration of infectiousness. Intuitively, a DENV-infected person with a longer duration of viremia has a greater chance of being bitten by, and infecting, a naïve *Aedes aegypti* mosquito. Therefore, patients with a high early viremia generally have a greater time-window of infectiousness for mosquitoes.

### Symptomatic vs. asymptomatic infections

Ambulatory patients with symptomatic DENV infections have viremia levels that are unquestionably likely to render them infectious to mosquitoes (32). Individuals who are asymptomatic with a DENV infection also have detectable levels of virus circulating in the blood (38, 39), but the question remains open as to whether or not inapparent DENV infections have high enough viremias to be infectious. Because inapparent DENV infections are common (40, 41), it follows that they could play a role in the maintenance of DENV in its natural transmission cycle, should their viremias be above the infectious threshold level.

### Understanding the extent of DENV transmission that is a result of asymptomatic infections

While the estimated number of asymptomatic DENV infections (over 290 million cases each year) outweighs that of symptomatic infections around the world (8) the contribution of these asymptomatic infections to the continued transmission of DENV remains to be elucidated. Definitive studies to determine whether acute asymptomatic cases are able to infect susceptible mosquitoes will give insight to the contribution of asymptomatic infections to the overall transmission dynamics. These can be done in two ways. The first involves more detailed surveillance and tracing of contacts of dengue index cases than that done in current longitudinal studies [e.g., those in Thailand (40), Nicaragua (41), and Peru (42)]. Increasing the frequency of blood draws of these contacts will help to identify asymptomatic cases at the earliest possible time. At first observation of viremia, the individual can be exposed to susceptible mosquitoes that are then tested for infection after a suitable incubation period.

Alternatively, we can gain this same knowledge in human challenge experiments, along with vast amounts of other information, on early infection dynamics (the portion of dengue pathogenesis that is least understood because patients only present to health care professionals after symptoms have already manifested). In human challenge studies, some participants will likely develop asymptomatic infections and the question of whether these individuals are infectious to mosquitoes can be tested in a controlled setting. In addition, such studies should also take such an opportunity to study the early infection dynamics in the participants in human challenge experiments with the aim in investigating the determinants of an infection becoming symptomatic or asymptomatic.

To date, there are few studies that have even demonstrated that asymptomatic infections result in a detectable viremia. Studies in both Nicaragua and Indonesia have described persons with acute asymptomatic DENV-1 and DENV-2 infections (having successfully amplified viral RNA by RT-PCR and/or by directly isolating the virus from the blood draw) using an index-case cluster surveillance approach described above (38, 39). This demonstrates it is indeed possible to study asymptomatic infections within the human host, but unfortunately in both studies, DENV viremia was not quantified, and mosquitoes were not exposed to the blood of these subjects, thus it is unknown if these individuals were infectious. Duong et al. (43) reported the first and only quantification of viremias in asymptomatic cases in the literature, however, these investigators did not assess infectiousness to mosquitoes. Until empirical evidence is obtained that supports the fact that mosquitoes can become infected, and infectious, after directly feeding on asymptomatic DENV infections, one cannot ascertain the extent to which these many millions of clinically silent infections are contributing to ongoing DENV transmission, or whether they are effectively dead-end hosts.

## Mosquito Susceptibility to Infection

Vector competence (VC) assays of mosquito susceptibility to DENV frequently test some combination of mosquito infection, dissemination, and onward transmission of virus. One factor potentially influencing our estimates of VC is that many studies have used artificially derived infectious blood meals to orally infect mosquitoes. In the first half of the twentieth century, mosquitoes were routinely fed on people suffering from dengue (33, 34, 36, 37, 44–48). When the weight of DENV research began to take place in non-endemic countries, a need for alternative methods arose. Since then, ordinarily, studies infect mosquitoes using artificial blood meals, consisting of a non-human blood source (often being derived from rabbit or pig), spiked with infectious virus grown in cell culture. While there are benefits of feeding mosquitoes using artificial blood meals (e.g., larger numbers of mosquitoes can be used, viral titers within the blood meal can be manipulated), employing the natural transmission mode to infect mosquitoes will help better describe the three-way human–mosquito–virus relationship in nature. Recognized factors influencing the VC of *Aedes aegypti* for DENV transmission are described below.

### Relative vector competence of *Aedes aegypti* and *Aedes albopictus*

Although *Aedes aegypti* are generally considered to be the primary vectors of DENV, *Aedes albopictus* have been implicated as the primary, if not the sole vector of DENV during some disease outbreaks (49, 50). Empirical studies show the two species do not differ in the competence to transmit DENV; both *Aedes aegypti* and *Aedes albopictus* collected from multiple sites within Cameroon showed no overall difference in their disseminated infection rate to DENV-2 (the same held true for infection with chikungunya virus also) (50). Similar results failing to identify differences in competence between the two species were reported for mosquitoes from the Florida Keys challenged with DENV-1 (51). Although both of these studies used artificial blood meals when infecting the mosquitoes and obtained similar results, the relative competence of these species after feeding on the viremic blood of a dengue case is unknown. A meta-analysis of 14 studies on the relative susceptibility of *Aedes albopictus* and *Aedes aegypti* suggests that *Aedes albopictus* are more susceptible to midgut infections than *Aedes aegypti*; however, the ability of the virus to disseminate in the latter mosquito is greater, suggesting a greater potential for transmission in nature (52).

### Virus concentration in the blood meal and the extrinsic incubation period

Numerous studies demonstrate that the proportion of mosquitoes that become infected with DENV depends on the concentration of virus in the blood meal (32, 53). Bennett et al. (53) identified a positive association between viral titer of DENV-2 in the infectious blood meal and the proportion of *Aedes aegypti* with an infected midgut. Once infected, however, rates of dissemination in the same mosquitoes showed no such association. As described in more detail above, in more than 200 patients with naturally acquired DENV infections, Nguyen et al. (32) detected a positive correlation between mosquito infection prevalence and the titer of virus in human blood (Figure 1), consistent across all four serotypes.

Viral titer can also influence the time that it takes for a mosquito to become infectious. Watts et al. (54) demonstrated that infecting Thai *Aedes aegypti* with a low titer of virus resulted in an extended period (up to 25 days) before the mosquitoes were able to transmit DENV-2 to naïve rhesus monkeys, compared to when using a higher titer of virus, where it took only 12 days after incubation at the same holding temperature of 30°C.

### Environmental temperature

Environmental temperature has long been implicated in altering mosquito VC to transmit viruses. A positive correlation between mean exposure temperature and the proportion of mosquitoes that become infected with the virus exists, that is bound by upper and lower limits (54–56). The lower the temperature, the longer it takes for the virus to replicate to high enough concentrations to be transmissible (and be detectable using laboratory techniques), but at high temperatures virus replication rates are greater, and the minimum time for the mosquito to complete the extrinsic incubation is decreased. Some populations differ in these values, but estimates for minimum and maximum thresholds for transmission (i.e., the temperature at which a mosquito can become infectious) at constant temperatures are around 13°C at the lower end (55), and 35°C at the upper end (54, 56) for *Aedes aegypti* mosquitoes (although higher temperatures are not known to have been tested). It is not known what these upper and lower limits are for *Aedes albopictus*.

Testing of the upper thresholds proves difficult, because after mosquitoes have been reared at such high temperatures (*cf*. 38°C and above) the lifespan of the mosquito is reduced due to the negative effect of the high heat; their flight activity is almost negligible and they are unable to source a blood meal (57). Therefore, assessing VC at such high temperatures must be performed at least in semi-unnatural conditions (offering the blood meal to the mosquitoes while at a cooler temperature and then placing them back at the exposure temperatures).

Several recent VC studies investigating transmission of mosquito-borne pathogens have also shown that using natural temperature exposures (ones that fluctuate throughout the day, as a mosquito experiences in nature as opposed to constant temperatures) can alter the expected VC of a mosquito population (56, 58–60). Reaction norms for VC (and other life-history traits) as characterized under constant temperatures failed to accurately predict the competence of *Aedes aegypti* for DENV transmission, after exposure to the same mean temperature, but with the addition of daily temperature fluctuations. Large fluctuations in the order of ~19°C around a low mean temperature of 20°C were shown to increase the number of *Aedes aegypti* that became infected with DENV-1, and accelerated the time that it took for dissemination to occur (by around 10 days) (56). Conversely, around a mean temperature of 26°C, one that is commonly used for laboratory-based experiments, the same magnitude of fluctuations had the opposite effect; there were fewer mosquitoes that developed a disseminated infection, and the first time dissemination observed was extended by 4 days (60). These studies highlight that it is important to empirically test mosquitoes under conditions representative of their natural environment to accurately measure VC used for modeling purposes.

Humidity changes may also play a role in mosquito VC, but precise measurements under variable humidity regimes have not been made. It is known that desiccation under dry conditions can place mosquitoes under stress. This stress may exacerbate the inability of the mosquito to fight off a viral infection, or indeed, may negatively impact the virus, because the mosquito may utilize available cellular resources for their maintenance before the virus has the opportunity to use them. At least in *Aedes albopictus*, changes in humidity can enhance the effect of changes in temperature affecting mosquito fecundity (61), and it follows that this is surmised to be the same in the closely related *Aedes aegypti*. More in-depth studies are required to elucidate the effect of humidity on VC indices.

### Population effects, and interactions between viral and mosquito genotypes

Populations of mosquitoes reportedly vary in their susceptibility to DENV infection (53, 62, 63), which can alter the accuracy of predictions of transmission dynamics among populations. On a large geographic scale, Gubler et al. (62) demonstrated population-specific differences in the ability of mosquitoes to become infected with each of the four DENV serotypes. Between populations, there were consistent patterns of high and low infection when exposed to each of the serotypes, suggesting that the factors controlling infection by each of the DENV serotypes is uniform and possibly conserved. Even on a relatively small scale, Bennett et al. (53) found that there was significant variation in the ability of 24 populations of mosquitoes from Mexico and USA to become infected with a DENV-2 strain.

There is also the suggestion that within a single population of mosquitoes, susceptibility to infection by different viral isolates/genotypes may vary (64–66). After challenging three isofemale lines of *Aedes aegypti* that were derived from Ratchaburi, Thailand, with three Thai isolates of DENV-1 virus (that were in current circulation), Lambrechts et al. (64) demonstrated that each of the Ratchaburi isofemale lines were most susceptible to infection by the viral isolate from the same city, Ratchaburi, as opposed to those from Kamphaeng Phet or Bangkok. A follow-on study identified polymorphisms at the *dicer-2* locus as being associated with these phenotypic differences in mosquito VC. Further studies demonstrating that this result holds true for mosquitoes derived from other populations are needed to show the generality of the phenomenon. In any case, the differences demonstrated between mosquito populations in their susceptibility to DENV infections suggest that mathematical models of DENV transmission need to consider the nuances of specific mosquito–virus interactions in their parameterization.

### Blood-feeding behavior and preferences of DENV mosquito vectors

One of the challenges standing in the way of developing targeted intervention approaches for the mosquito to human transmission cycle include a lack of understanding of mosquito behaviors, including that of host-seeking. A cornerstone of the DENV transmission cycle is the mosquito vector, and without an infected mosquito’s success in seeking a suitably DENV-naïve host, transmission would cease and the virus would die. Since other bacteria and viruses manipulate the biology and behavior of their hosts to facilitate their own transmission (67, 68), it is plausible that DENV may do the same. Studies on the blood-feeding behavior of DENV-infected mosquitoes have examined duration of probing and feeding (69, 70), transmission efficiency during probing (71), and motivation and avidity to feed (72). While DENV infections may increase the duration of feeding and the likelihood of re-feeding after interruption (as tested using either mice or guinea pigs), no studies have directly investigated human host-seeking ability.

Hypothetically, if an uninfected mosquito is potentially attracted to human hosts with a high body temperature (e.g., as a result of fever), does DENV then manipulate the physiology of an infected mosquito to be more attracted to people with lower body temperature (e.g., those that are likely to be uninfected) for their subsequent meals, to increase the likelihood of transmission? Can DENV increase the frequency and/or desire to blood feed, leading to mosquito vectors feeding on multiple hosts, thereby enhancing transmission? Finally, does the virus alter the physiology of the human host (other than causing high fever) in ways that are detectable to a mosquito, making them more attractive? Investigating the host-feeding preferences and host-seeking ability of infected and uninfected mosquitoes can help elucidate the extent to which DENV manipulates its mosquito vectors.

### Other factors influencing infection

Mosquitoes have an increased risk of infection when exposed to naturally infected dengue patients when they have a high tympanic temperature and high plasma viremia (32). With the progression of illness in a patient, IgM and IgG titers continue to increase until after viremia declines beyond a detectable limit. With this increasing day of illness and associated IgM and IgG titers, *Aedes aegypti* experience a decreasing risk of DENV infection (32). Increased titers of these antibodies in the blood may directly influence mosquito susceptibility, by neutralizing virus and preventing infection of the midgut.

### Novel entomological strategies for reducing DENV transmission in the field

A number of novel strategies are being developed that control mosquito populations. These include but are not limited to the use of genetic manipulations of mosquitoes, fungus, and bacteria to curb pathogen transmission. The RIDL (release of insects carrying a dominant lethal) technique releases genetically modified males into a mosquito population that carry a late-acting lethal, development gene that is transmitted to each of its progeny (73). Fungal biopesticides have also been proposed for control of mosquito transmission of pathogens (74).

Another of these strategies intends to release mosquitoes infected with the intracellular bacterium *Wolbachia pipientis* (75, 76). In *Aedes aegypti, Wolbachia* manipulate the host reproduction system to enhance its own vertical transmission between generations, but can also reduce host lifespan (77), and critically interfere with DENV replication (78). The level of virus interference in *Aedes aegypti* is however dependent on the bacterial strain.

Releasing mosquitoes into the wild that contain this intracellular bacterium aims to reduce the ability of mosquitoes to transmit DENV under field conditions. After the initial establishment phase of the release, this biological control strategy is self-maintained due to *Wolbachia*’s ability to drive itself into a population of hosts, thereby increasing the benefit of this strategy by decreasing long-term maintenance costs. Additionally, the technology can be implemented relatively cheaply, meaning that countries that face a large dengue burden may see the greatest value in its implementation. There are also multiple strains of the bacteria that can be utilized, with different incompatibility phenotypes, offering the opportunity for multiple releases. Field releases of *Wolbachia*-infected *Aedes aegypti* have already occurred in Northern Australia, Vietnam, and Indonesia with the aim of suppressing DENV transmission.

One theoretical concern about this strategy is the long-term efficacy of the program due to evolutionary changes in the genomes of vector, virus, and/or bacteria. Evolution may erode the viral replication inhibition effect of the bacteria, increased virulence of the virus in humans, and decrease the life-shortening phenotype in the bacterial host, as seen in the native Drosophila host of the life-shortening *Wolbachia* strain (79). An objective discussion of the potential evolutionary changes in the *Wolbachia* vs. DENV relationship, within the human–*Aedes aegypti* framework, is presented by Bull and Turelli (80).

## Summary and Research Priorities

The successful transmission of DENV from human to mosquitoes is a complex interplay of biotic and abiotic factors. Despite this, DENV transmission occurs on a global scale and continues to be the most prevalent arbovirus infection, with an estimated 390 million infections each year (8). At this point, there are several research priorities that would benefit our understanding of human to mosquito transmission, and subsequently aid research and development into the long-term goal of finding effective tools for DENV prevention (e.g., vaccines, prophylactic or therapeutic use of antivirals, and vector control). These research priorities are:

(1) To what extent do asymptomatic infections contribute to ongoing transmission? What proportion of asymptomatic infections result in mosquitoes being capable of transmission? What is the range of viremia required for transmission to occur?
(2) Can antivirals and/or neutralizing antibodies be administered to dengue patients to reduce the potential for DENV patients to infect naïve mosquitoes? Can antibodies neutralize the virus in the mosquito before it becomes infected? How would this feasibly be administered?
(3) How will dengue vaccines modify viremia after natural exposure? Will they modify viremia to a level that prevents human to mosquito transmission?
(4) What preferences do *Aedes* mosquitoes show toward febrile and non-febrile hosts? Are naïve mosquitoes more attracted to febrile hosts (infected with any arbovirus)? Does DENV manipulate host-seeking behavior in infected mosquitoes?
(5) Can the likely field success of novel dengue control measures, such as *Wolbachia*, be predicted from laboratory studies? Which of the many and complex effects of *Wolbachia* on *Aedes aegypti* life-history traits have the greatest impact on VC?

Advances in our understanding of the DENV transmission cycle in humans and mosquitoes should support the rational development and application of interventions such as vaccines, antivirals, and novel entomological control measures.

## Author Contributions

Lauren B. Carrington and Cameron P. Simmons conceived and wrote the manuscript.

## Conflict of Interest Statement

Cameron P. Simmons is a consultant to Merck and Sanofi Pasteur for the development of dengue vaccines, and to GSK, Unither Virology, and Tibotec for the development of anti-viral therapies
